# Accuracy and depth evaluation of clinical low pass genome sequencing in the detection of mosaic aneuploidies and CNVs

**DOI:** 10.1186/s12920-023-01703-8

**Published:** 2023-11-17

**Authors:** Yanqiu Liu, Shengju Hao, Xueqin Guo, Linlin Fan, Zhihong Qiao, Yaoshen Wang, Xiaoli Wang, Jianfen man, Lina Wang, Xiaoming Wei, Huanhuan Peng, Zhiyu Peng, Yan Sun, Lijie Song

**Affiliations:** 1 Jiangxi Maternal and Child Health Hospital Affiliated to Nanchang Medical College, Nanchang, 33000 Jiangxi China; 2 Medical Genetics Center, Gansu Provincial Clinical Research Center for Birth Defects and Rare Diseases, Gansu Provincial Maternity and Child-care Hospital, Lanzhou, 730050 China; 3 Clin Lab, BGI Genomics, Wuhan, 430074 China; 4 Clin Lab, BGI Genomics, Tianjin, 300308 China; 5 Clin Lab, BGI Genomics, Shenzhen, 518083 China; 6 MGI Tech, Shenzhen, 518083 China; 7 BGI Genomics, Shenzhen, 518083 China; 8 DTU Bioengineering, Technical University of Denmark, Kongens Lyngby, 2800 Denmark

**Keywords:** Low pass genome sequencing, Mosaic CNVs, Mosaic aneuploidies

## Abstract

**Background:**

Low-pass genome sequencing (LP GS) has shown distinct advantages over traditional methods for the detection of mosaicism. However, no study has systematically evaluated the accuracy of LP GS in the detection of mosaic aneuploidies and copy number variants (CNVs) in prenatal diagnosis. Moreover, the influence of sequencing depth on mosaicism detection of LP GS has not been fully evaluated.

**Methods:**

To evaluate the accuracy of LP GS in the detection of mosaic aneuploidies and mosaic CNVs, 27 samples with known aneuploidies and CNVs and 1 negative female sample were used to generate 6 simulated samples and 21 virtual samples, each sample contained 9 different mosaic levels. Mosaic levels were simulated by pooling reads or DNA from each positive sample and the negative sample according to a series of percentages (ranging from 3 to 40%). Then, the influence of sequencing depth on LP GS in the detection of mosaic aneuploidies and CNVs was evaluated by downsampling.

**Results:**

To evaluate the accuracy of LP GS in the detection of mosaic aneuploidies and CNVs, a comparative analysis of mosaic levels was performed using 6 simulated samples and 21 virtual samples with 35 M million (M) uniquely aligned high-quality reads (UAHRs). For mosaic levels > 30%, the average difference (detected mosaic levels vs. theoretical mosaic levels) of 6 mosaic CNVs in simulated samples was 4.0%, and the average difference (detected mosaic levels vs. mosaic levels of Y chromosome) of 6 mosaic aneuploidies and 15 mosaic CNVs in virtual samples was 2.7%. Furthermore, LP GS had a higher detection rate and accuracy for the detection of mosaic aneuploidies and CNVs of larger sizes, especially mosaic aneuploidies. For depth evaluation, the results of LP GS in downsampling samples were compared with those of LP GS using 35 M UAHRs. The detection sensitivity of LP GS for 6 mosaic aneuploidies and 15 mosaic CNVs in virtual samples increased with UAHR. For mosaic levels > 30%, the total detection sensitivity reached a plateau at 30 M UAHRs. With 30 M UAHRs, the total detection sensitivity was 99.2% for virtual samples.

**Conclusions:**

We demonstrated the accuracy of LP GS in mosaicism detection using simulated data and virtual samples, respectively. Thirty M UAHRs (single-end 35 bp) were optimal for LP GS in the detection of mosaic aneuploidies and most mosaic CNVs larger than 1.48 Mb (Megabases) with mosaic levels > 30%. These results could provide a reference for laboratories that perform clinical LP GS in the detection of mosaic aneuploidies and CNVs.

**Supplementary Information:**

The online version contains supplementary material available at 10.1186/s12920-023-01703-8.

## Introduction

Mosaicism refers to the presence that an individual contains more than one clone of cells, which are derived from a single fertilized egg but with distinct genotypes [1]. Many disorders, such as developmental delay, congenital anomalies and miscarriage, have been reported to be related to mosaicism [2]. Depending on the proportion of chromosomes or alleles and cells with the variant, the pathogenicity, risk, and outcome of mosaic varied a lot [2]. In a review of 660 cases of autosomal mosaic trisomy detected in amniocytes from prenatal diagnosis, the risk of abnormal outcome was summarized to improve the data available for genetic counseling [3]. To provide a precise diagnosis and risk analysis for genetic counseling, an assay for rapid and accurate mosaicism detection is in need.

Karyotyping is recognized as the gold standard assay for the detection of aneuploidies, microdeletions and duplications, and mosaicism of these variants [4, 5]. Other assays, such as fluorescence in situ hybridization (FISH) and quantitative fluorescent PCR (QPCR) also played an important role in the detection of mosaicism [6, 7]. In recent years, new assays with higher throughput and resolution were developed, such as chromosomal microarray analysis (CMA) and low pass genome sequencing (LP GS, also known as CNV-seq) [8–10]. These assays offered distinct advantages over traditional methods for the detection of mosaicism. LP GS is performed on uncultured cells, therefore eliminating the detection of pseudo mosaicism caused by cultural artifacts [11]. LP GS also showed greater potential for the detection of low-level mosaicism compared with CMA [12].

However, no study has systematically evaluated the accuracy of LP GS in the detection of mosaic aneuploidies and CNVs. It has been reported that the levels of mosaicism of aneuploidies were different using karyotyping, CMA and LP GS [10, 13]. Although LP GS detected more mosaicism (most of which were low level mosaicism), the accuracy was still unknown. What is more, the influence of sequencing depth on LP GS in the detection of mosaic aneuploidies and CNVs has not been fully evaluated. In genomic analyses, the number of UAHRs (DP) is a crucial factor and key consideration for MPS-based technology. The sequencing depth of LP GS is a key factor that has not been comprehensively studied. These things still need to be systematically investigated before LP GS can be used as a first-tier genetic test for the detection of mosaic aneuploidies and CNVs in prenatal diagnosis. The expected mean DP represents the average number of times that a base is covered by high-quality aligned reads [14]. To facilitate calculation and demonstration, in this study we used the number of UAHRs to perform DP evaluation.

Regarding the range of mosaicism (percentage of abnormal cells) used to determine an aneuploid embryo, euploid embryo or mosaic embryo, some groups suggested a typical cut-off level of ≤ 20% for euploid assignment and ≥ 80% for aneuploidy assignment, while some groups used a cut-off level of ≤ 30% for euploid assignment and ≥ 70% for aneuploidy assignment [15, 16]. For LP GS, a study suggested a lower cut-off value of 20% mosaicism for clinical reporting of fetal aneuploidies and unbalanced chromosomal structural abnormalities in prenatal diagnosis in China [17].

In this study, we comprehensively evaluated the accuracy and benchmarked the optimal DP of LP GS in the detection of mosaicism. We demonstrated the accuracy of LP GS in the detection of mosaic aneuploidies and CNVs using 6 simulated data and 21 virtual samples with the same read amounts, respectively. We also found that CNV size influenced the accuracy of LP GS in the detection of mosaic aneuploidies and CNVs. Then, we performed a depth evaluation of LP GS in mosaicism detection. The results showed that the detection sensitivity of LP GS for mosaic aneuploidies and CNVs was influenced by UAHR. As a result, the number of 30 M UAHRs was recommended for the detection of mosaic aneuploidies and identification of most mosaic CNVs with mosaic levels > 30%. These results could provide a reference for laboratories to perform clinical LP GS in mosaicism detection.

## Methods

### Study design and sample collection

**Fig. 1:**
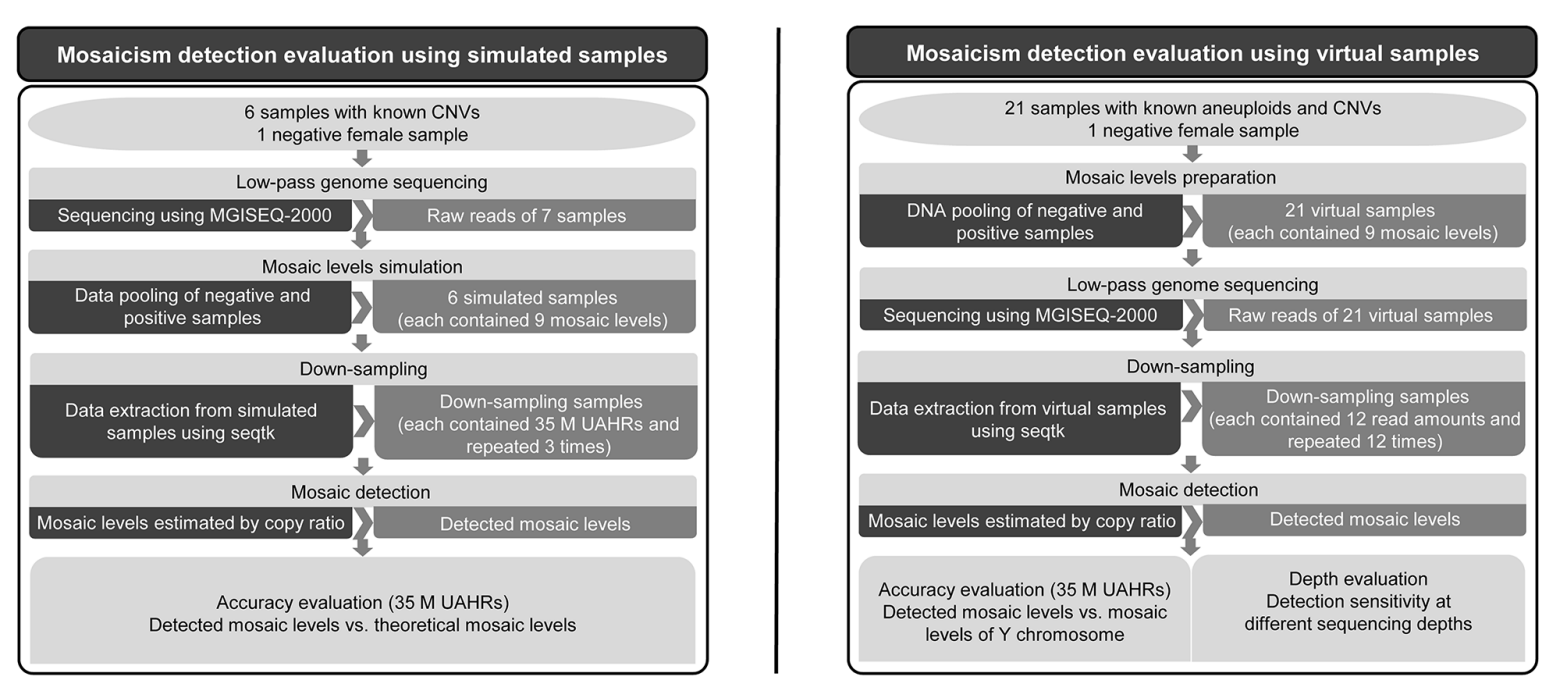
Study design

To evaluate the accuracy and benchmark the optimal DP of LP GS in the detection of mosaicism, evaluation strategies were designed using simulated data and virtual samples, respectively (Fig. 1). The DNA of a total of 28 clinical samples (S1 ~ S28) (aborted fetal tissue, whole blood, chorionic villus and amniotic fluid) were included in this study (Supplementary Table S1). S1 ~ S27 were positive samples with 27 known pathogenic aneuploidies and CNVs (6 aneuploidies, 15 deletions and 6 duplications). S28 was a negative female sample. Informed consent was obtained from all the participants. All informed consent forms indicate that the samples can be used for scientific research after removing personally identifiable information. This study and all the protocols followed herein were approved by the Institutional Review Board of BGI (NO. BGI-IRB 22062).

### LP GS

LP GS (single-end 35 bp) of the 28 samples (S1 ~ S28) and 21 virtual samples prepared from these samples was performed as described in previous studies using MGISEQ-2000 platform [18–20].

Bioinformatics analysis was performed on 6 simulated samples, 21 virtual samples, and down-sampling samples from both simulated and virtual samples. In general, after initial quality control, reads were aligned to SOAP2 to generate UAHRs. For CNV detection, the optimum window size was 50 K bp. First, a two-step correction procedure was performed to remove the local GC content bias and multiplex-related bias, and an unbiased relative copy ratio for each window was generated using UAHRs, then the candidate CNV breakpoints were identified using a binary segmentation method, and finally a combined statistics test using U-test and Parallelism-test was recruited to determine CNV genotypes and filter out false positives [18–20]. Mosaic levels of mosaic aneuploidies and mosaic CNVs were estimated by the differences of observed copy ratio (${R}_{observed}$) compared with a normal copy ratio (${R}_{CNV}$). Mosaic levels of mosaic aneuploidies and mosaic CNVs were denoted by the following formulas:

1 $${P_{mosaicdeletion(chr1\, \sim chr23)}} = \frac{{1 - {R_{observed}}}}{{1 - {R_{CNV}}}} \cdot {R_{CNV}} = 0.5$$

2 $${P_{mosaicduplication(chr1 \sim chr23)}} = \frac{{{R_{observed}} - 1}}{{{R_{CNV}} - 1}} \cdot {R_{CNV}} = 1.5$$

3 $${P_{mosaicism\,\left( {chrY} \right)}} = \frac{{{R_{observed}}}}{{{R_{CNV}}}} \cdot {R_{CNV}} = 1$$

### Mosaicism detection evaluation using simulated samples

Six (S1 ~ S6) positive samples (6 known CNVs) and 1 negative female sample (S28) were collected to generate 6 simulated samples (Fig. 1). The 6 known CNVs ranged in size from 1.62 Mb to 9.88 Mb, involving 4 diseases (Supplementary Table S1). After LP GS of the 7 samples, 9 different mosaic levels were simulated for each positive sample. Mosaic levels were simulated by pooling reads from each positive sample and the negative sample according to a series of percentages (3%, 5%, 10%, 15%, 20%, 25%, 30%, 35%, 40%) using seqtk (https://github.com/lh3/seqtk). First, the total number of reads (${n}_{total}$) of the negative sample was calculated. Then, the number of reads required from the positive sample (${n}_{positive}$) and the negative sample (${ n}_{negative}$) to simulate a mosaic level (${mosaic}_{s}$) is calculated by the following formula:

1 $${n}_{positive}={n}_{total}*{mosaic}_{s}$$

2 $${n}_{negative}={n}_{total}-{n}_{positive}$$

For accuracy evaluation, UAHR was used to generate down-sampling samples for 6 simulated samples, each contained 9 different theoretical mosaic levels (3%, 5%, 10%, 15%, 20%, 25%, 30%, 35%, 40%) (Fig. 1). For each simulated sample at a certain mosaic level, random downsampling using seqtk (https://github.com/lh3/seqtk) was performed to generate35 M UAHRs. For each sample (each contained 9 mosaic levels) with 35 M UAHRs, random down-sampling were repeated 3 times.

It was reported that CNVs larger than 751 kb were readily detected at 30% mosaic level with a read-depth of about 0.25X [12]. To further study the detection accuracy of LP GS for mosaicism, we increased the depth to 35 M UAHRs (equivalent to a read-depth of 0.41-fold).

To evaluate the accuracy of LP GS in the detection of mosaic CNVs, a comparative analysis of the detected mosaic levels at 35 M UAHRs and the theoretical mosaic levels was performed for 6 mosaic CNVs (Supplementary methods). In addition, we also compared the mosaic levels of Y chromosome and the theoretical mosaic levels for 4 mosaic CNVs in 4 male simulated samples (Supplementary methods).

#### Mosaicism detection evaluation using virtual samples

Twenty-one (S7 ~ S27) positive male samples (known 6 aneuploidies and 15 CNVs) and 1 negative female sample (S28) were collected to prepare 21 virtual samples (Fig. 1). We selected male samples to verify the success of the experiment by comparing the mosaic levels of Y chromosome with the theoretical mosaic levels. The 6 aneuploidies and 15 CNVs ranged in size from 1.48 Mb to 115.17 Mb, involving 21 diseases (Supplementary Table S1). After LP GS of the 22 samples, 9 different mosaic levels were simulated for each positive sample. Mosaic levels were simulated by pooling the DNA from each positive sample and the negative sample according to a series of percentages (3%, 5%, 10%, 15%, 20%, 25%, 30%, 35%, 40%). First, each sample was diluted to the same concentration and the required DNA amount from positive sample (${m}_{positive}$) was determined. Then, the required DNA amount from the negative sample (${m}_{negative}$) was computed by the following formula:

3 $${m}_{negative}={m}_{positive}\frac{1-{mosaic}_{s}}{{mosaic}_{s}}$$

For accuracy and depth evaluation, down-sampling samples for 21 virtual samples were used, each contained 9 different theoretical mosaic levels (3%, 5%, 10%, 15%, 20%, 25%, 30%, 35%, 40%). For each virtual sample at a certain mosaic level, random downsampling was performed to generate 12 different sequencing depths (target numbers of the UAHRs: 100 K, 300 K, 500 K, 1 M, 3 M, 5 M, 10 M, 15 M, 20 M, 25 M, 30 M, 35 M) using seqtk (https://github.com/lh3/seqtk). For each sample (each contained 9 mosaic levels) with a certain UAHR, random down-sampling were repeated 12 times.

For simulated samples, 3 downsampling samples with 35 M UAHRs were used for the accuracy evaluation. For virtual samples, 3 downsampling samples with the same sequencing depth (35 M UAHRs) were randomly selected to evaluate the detection accuracy. In addition, we used the results of 3 downsampling samples with 35 M UAHRs as a reference standard for comparison with the results after downsampling to ensure that the depth evaluation was performed under the same standard.

To evaluate the detection accuracy of LP GS in the detection of mosaic aneuploidies and CNVs, a comparative analysis of the detected mosaic levels, the mosaic levels of Y chromosome, and the theoretical mosaic levels was performed for 6 mosaic aneuploidies and 15 mosaic CNVs (Supplementary methods).

For depth evaluation of LP GS in the detection of mosaic aneuploidies and CNVs, the results of LP GS in downsampling samples were compared with those of LP GS using 35 M UAHRs (Supplementary methods).

## Results

### Accuracy evaluation of LP GS using simulated samples

**Fig. 2:**
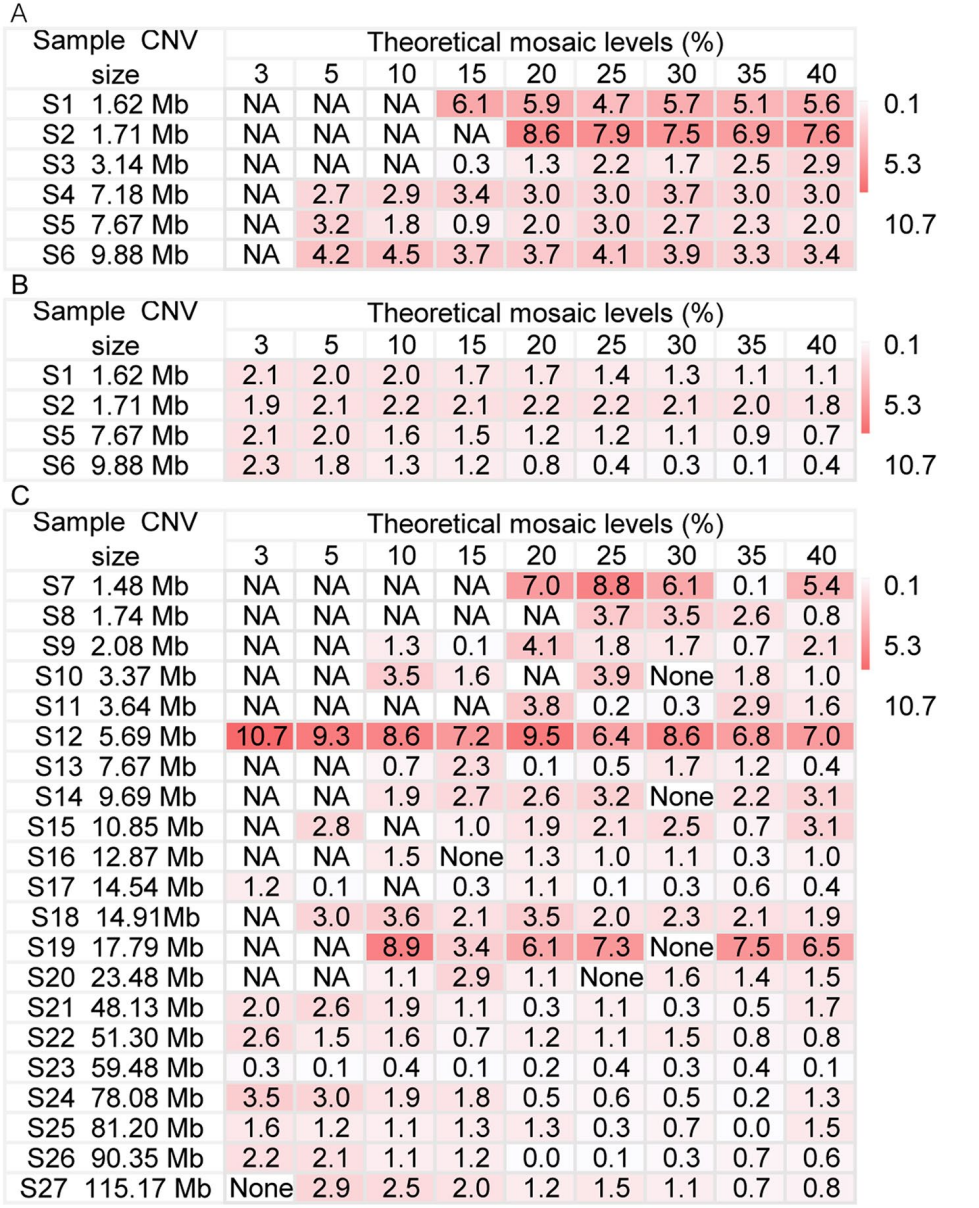
Accuracy evaluation of LP GS in the detection of mosaic aneuploidies and CNVs in simulated samples and virtual samples. **(A)** Differences (%) between the detected mosaic levels and the theoretical mosaic levels for 6 mosaic CNVs in 6 simulated samples. **(B)** Differences (%) between the mosaic levels of Y chromosome and the theoretical mosaic levels for 4 mosaic CNVs in 4 male simulated samples. **(C)** Differences (%) of the detected mosaic levels and the mosaic levels of Y chromosome for 6 mosaic aneuploidies and 15 mosaic CNVs in 21 virtual samples. ‘NA’ shows that the mosaic level was not detected in at least one of 3 downsampling samples at 35 M UAHRs.‘None’ shows that the number of UAHRs of the sample is less than 35 M.

To evaluate the accuracy of LP GS in the detection of mosaic CNVs, we performed computational simulations using 6 positive samples and a female negative sample to generate 6 simulated samples (6 mosaic CNVs), each sample contained 9 different mosaic levels. About an average of 62.20 M UAHRs (single-end 35 bp) were obtained for each simulated sample (Table S2). The differences between the detected mosaic levels and the theoretical mosaic levels were used to evaluate the accuracy of LP GS in the detection of mosaic CNVs. The results of simulated samples with 35 M UAHRs were shown in Fig. 2A. For 6 mosaic CNVs with mosaic levels > 30% (the cutoff level for euploid assignment suggested by published studies [15, 16]), the average difference between the detected mosaic levels and the theoretical mosaic levels was 4.0%. In addition, LP GS could not detect low mosaic levels ranging from ~ 3% to ~ 10% for 3 CNVs ranging in size from 1.62 Mb to 3.14 Mb. For 2 mosaic CNVs ranging in size from 1.62 Mb to 1.71 Mb, the detection rate was 61.1% (11/18), the average difference in mosaic levels was 6.5%. For 4 mosaic CNVs ranging in size from 3.14 Mb to 9.88 Mb, the detection rate was 91.7% (30/36), the average difference in mosaic levels was 2.8%. The results indicated that LP GS had a higher detection rate and accuracy for mosaic CNVs with larger CNV size.

In addition, we also compared the detected mosaic levels of Y chromosome and the theoretical mosaic levels for 4 male simulated samples (S1, S2, S5, S6) with 35 M UAHRs. The results showed that the average differences between the mosaic levels of Y chromosome and the theoretical mosaic levels were 1.5% (Fig. 2B). Moreover, for the 4 mosaic CNVs, the differences between the mosaic level of Y chromosome and the theoretical mosaic levels were smaller than the corresponding differences between the detected mosaic levels and the theoretical mosaic levels (Fig. 2A and B). Therefore, we used the difference between the mosaic level of Y chromosome and the theoretical mosaic level as the benchmark to verify the success of the experiment for the preparation of various mosaic levels. For low-level mosaicism, such as 3%, it was difficult for an experiment to have a very small error such as 1%, which leads to the difference between the actual mosaic level and the theoretical mosaic level. So, it was hard to accurately prepare low mosaic levels by experimental methods. For virtual samples, the mosaic levels of Y chromosome were used for comparison analysis in accuracy evaluation.

### Accuracy evaluation of LP GS using virtual samples

To evaluate the accuracy of LP GS in the detection of mosaic aneuploidies and CNVs, 21 virtual samples (6 mosaic aneuploidies and 15 mosaic CNVs) were prepared at 9 different mosaic levels. An average of 60.28 M UAHRs (single-end 35 bp) were obtained for virtual samples (Table S2). The differences between the detected mosaic levels and the mosaic levels of Y chromosome were used to evaluate the accuracy of LP GS in the detection of mosaic aneuploidies and CNVs. The results of virtual samples with 35 M UAHRs were shown in Fig. 2C. For 6 mosaic aneuploidies and 15 mosaic CNVs with mosaic levels > 30%, the average difference between the detected mosaic levels and the mosaic levels of Y chromosome was 1.8%. In addition, for 14 mosaic CNVs ranging in size from 1.48 Mb to 23.48 Mb, the detection rate was 73.6% (89/121), the average difference in mosaic levels was 2.9%. For a mosaic CNV and 6 mosaic aneuploidies ranging in size from 48.13 Mb to 115.17 Mb, the detection rate was 100% (62/62), and the average difference in mosaic levels was 1.1%. The results indicated that LP GS had a higher detection rate and accuracy for mosaic aneuploidies and CNVs with larger CNV size, especially for mosaic aneuploidies.

### Depth evaluation of LP GS using virtual samples

**Fig. 3:**
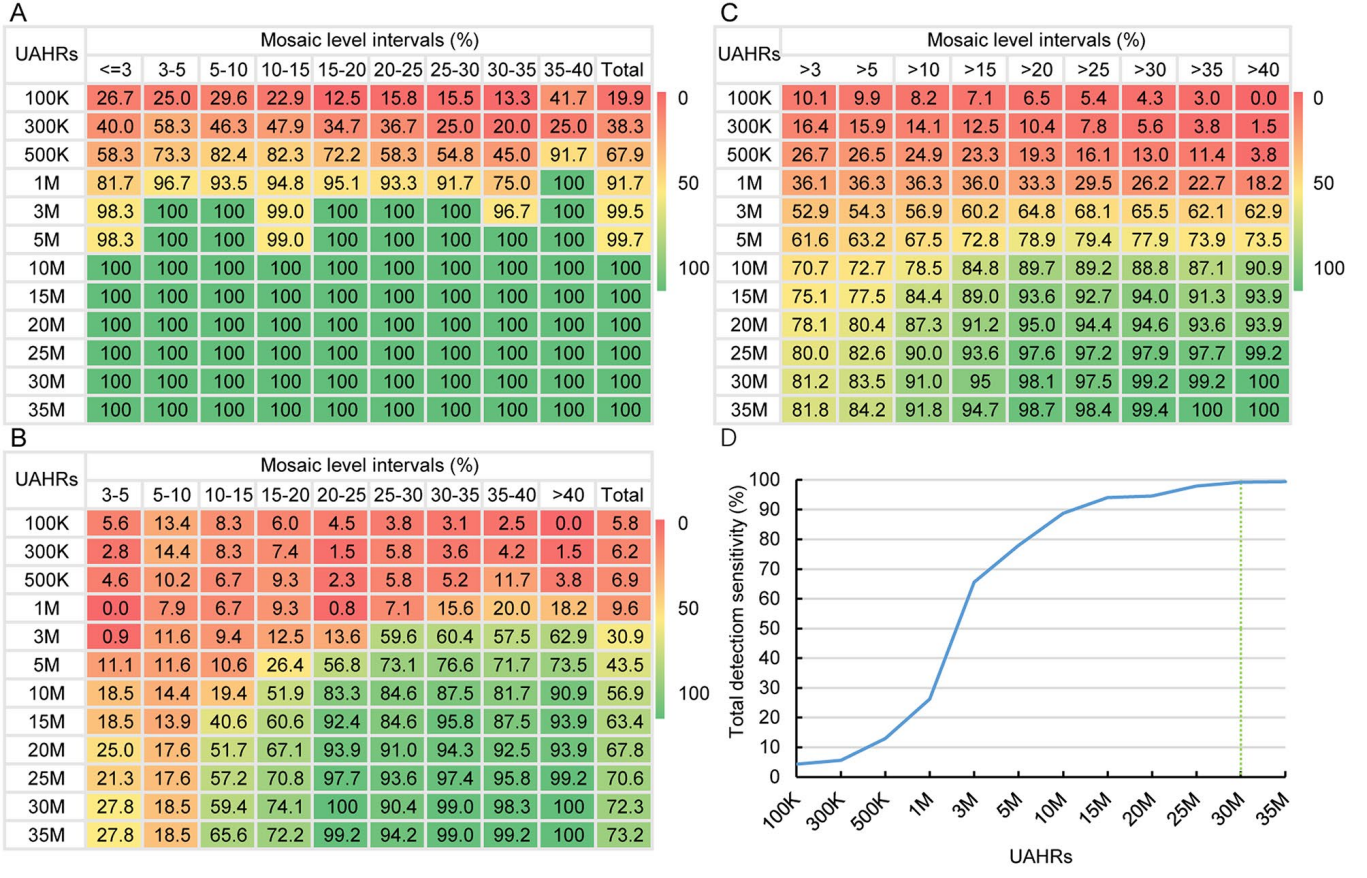
Depth evaluation of LP GS in the detection of mosaic aneuploidies and CNVs in virtual samples. **(A)** Detection sensitivity (%) of LP GS for 6 mosaic aneuploidies in different mosaic level intervals at each sequencing depth. (B) Detection sensitivity (%) of LP GS for 15 mosaic CNVs in different mosaic level intervals at 12 different sequencing depths. **(C)** Detection sensitivity (%) of LP GS for 6 mosaic aneuploidies and 15 mosaic CNVs in different mosaic level intervals at 12 different sequencing depths. **(D)** Detection sensitivity of LP GS for 6 mosaic aneuploidies and 15 mosaic CNVs with mosaic levels > 30% at 12 different sequencing depths using virtual samples. The dotted green line shows the number of UAHRs (30 M) when the detection sensitivity reached a plateau

To evaluate the influence of sequencing depth on LP GS for mosaic aneuploidies and CNVs, UAHRs were used to generate downsampling samples for 6 mosaic aneuploidies and 15 mosaic CNVs. The results showed that the detection sensitivity of LP GS for mosaic aneuploidies and CNVs increased with UAHR (Fig. 3A and B). For mosaic levels > 30%, the total detection sensitivity reached a plateau at 30 M UAHRs. With 30 M UAHRs, the total detection sensitivity was 99.2% for 6 mosaic aneuploidies and 15 mosaic CNVs (Fig. 3C and D).

## Discussion

In our study, we performed accuracy and depth evaluation of LP GS in the detection of mosaic aneuploidies and CNVs using simulated data and virtual samples, respectively. First, we demonstrated the accuracy of LP GS in mosaicism detection using simulated samples and virtual samples, respectively. We also found that CNV size influenced the accuracy of LP GS in the detection of mosaic aneuploidies and CNVs. LP GS had a higher detection rate and accuracy for mosaic aneuploidies and CNVs with larger CNV size, especially for mosaic aneuploidies. In addition, we performed depth evaluation of LP GS on mosaicism detection. The results showed that the detection sensitivity of LP GS for mosaic CNVs was influenced by UAHR. As a result, the number of 30 M UAHRs (single-end 35 bp) were recommended for the detection of mosaic aneuploidies and most mosaic CNVs larger than 1.48 Mb with mosaic levels > 30%.

The setting (mosaic CNV detection) closely mirrors CNV detection in non-invasive prenatal testing (NIPT), where “mosaicism” is inherently present due to the fraction of fetal DNA. We compared the findings with 2 previous studies regarding CNV detection in the NIPT setting. Using LP GS (approximately 0.2-fold), a simulation study (17.5%, 15%, 12.5%, 10%, 7.5%, and 5% mixtures) demonstrated that the sensitivity difference between their method and the theoretical limit was < 5% for MDs ≥ 9 Mb [21]. In our study, for 5% mosaic level, with 20 M UAHRs (approximately 0.23-fold), the total detection sensitivity was 61.4% for mosaic MDs ≥ 9.69 Mb. An in-silico study showed sensitivity of 79.3% for 10% fetal fraction with 20 M UAHRs (2 × 35 bp, approximately 0.47-fold), which further increased to 98.4% if only for deletions longer than 3 Mb [22]. In our study, for 10% mosaic level, with 35 M UAHRs (approximately 0.41-fold), the total detection sensitivity was 85.4% for mosaic aneuploidies and CNVs ≥ 3.37 Mb. Comparing with the 2 studies in NIPT setting [21, 22], the detection sensitivity in our study was lower. The differences in detection sensitivity may be due to different methods and sample sets for testing. For example, 45 gDNA mixture models were created simulate 7 mixtures (20%, 17.5%, 15%, 12.5%, 10%, 7.5%, 5%), and methods for removing sequencing bias (data normalization), localizing the CNV breakpoints (circular binary segmentation), and combating false positives (decision tree) were used to detect aneuploidies or microdeletion/microduplications (MDs). In our study, 6 simulated samples and 21 virtual samples were used to simulate 9 different mosaic levels (3%, 5%, 10%, 15%, 20%, 25%, 30%, 35%, 40%), and similar CNV detection methods were used. However, the 45 samples only involved four diseases (DiGeorge, Cridu-chat, Prader-Willi, Angelman, and 1p36 deletion syndromes). The position of CNV regions was an important factor affecting detection sensitivity [21]. Sensitivity for only 4 regions may be biased. In addition, some details of the specific methods were different, such as statistical methods, z-score was used in the study, while U-test and Parallelism-test were used in our study. Therefore, different methods and sample sets may yield different sensitivities.

We compared the findings with a study regarding CNV detection in the mosaic setting [12]. A data simulation study demonstrated that 15 M reads (single-end 50 bp, approximately 0.25-fold) were required for 30% mosaic level to be readily detected, and CNVs larger than 2.5 Mb are also detectable at mosaic levels as low as 20% [12]. In our study, for 30% mosaic level, with 20 M UAHRs (approximately 0.29-fold), the total detection sensitivity was 98% for mosaic aneuploidies and CNVs ≥ 1.48 Mb. For 20% mosaic level, with 20 M UAHRs (approximately 0.29-fold), the total detection sensitivity was 99.5% for mosaic aneuploidies and CNVs ≥ 5.69 Mb. Comparing with the study [12], despite the differences in methods and sample sets, the result was not significantly different and was basically consistent.

For simulated samples, the accuracy of LP GS in the detection of mosaic aneuploidies and CNVs was evaluated by the difference between the detected mosaic levels and the theoretical mosaic level. However, for virtual samples, the accuracy of LP GS in the detection of mosaic aneuploidies and CNVs was evaluated by the differences between the detected mosaic levels and the mosaic levels of Y chromosome. As mentioned in the accuracy evaluation using simulated samples, for 4 mosaic CNVs in 4 male simulated samples, the differences between the mosaic levels of Y chromosome and the theoretical mosaic levels were smaller than the corresponding differences between the detected mosaic levels and the theoretical mosaic level (Fig. 2A and B). So only male samples were selected to prepare virtual samples to verify the success of the experiment by comparing the mosaic levels of Y chromosome with the theoretical mosaic levels. We found that the differences between the mosaic levels of Y chromosome and the theoretical mosaic levels fluctuated greatly in virtual samples (Supplementary figure S1). It was difficult to obtain a small difference between the mosaic level of experimental preparation and the theoretical mosaic level, especially for low-level mosaicism. Therefore, for virtual samples, the mosaic level of Y chromosome was used for comparison analysis in accuracy evaluation.

For virtual samples, we found that some differences between the detected mosaic levels and the mosaic levels of Y chromosome were relatively large, especially sample S12. Sample S12 (Prader-Willi syndrome/Angelman syndrome) with a mosaic CNV (del15q11.2q13.1, 5.69 Mb) had the largest average difference of 8.2% among the 21 virtual samples (Fig. 2C). It could provide a reference for laboratories using clinical LP GS in the detection of mosaic deletions in region 15q11.2q13.1. The reason for the large mosaic level difference in this region needs further study.

The reasons for using pathogenic aneuploidies and CNVs for accuracy and depth evaluation of LP GS in this study: (1) For symptoms of aneuploidies and CNVs, it was more significant to study pathogenic aneuploidies and CNVs than non-pathogenic aneuploidies and CNVs; (2) There were no systematic reviews of studies performing risk prediction for mosaic CNVs, and only sporadic reports of a CNV at a mosaic level had clinical symptoms. Therefore, the mosaic aneuploidies and mosaic CNVs in the positive samples selected in this study were all pathogenic.

The minimum mosaic level tested in this study was ~ 3%. LP GS can be used for the detection of low-level mosaicism. But it has been reported that the low-level mosaicism has lower pathogenicity [13], which may be less meaningful for the study of lower mosaic levels, and it is difficult to prepare such low mosaic levels for experiments. Low-level mosaicism has different risks for different types of variants. It was reported that low-level mosaic aneuploidy was a genetic risk factor for autism [23], schizophrenia pathogenesis [24], and neurodevelopmental disorders [20]. Low-level mosaic CNVs were reported in several conditions such as holoprosencephaly [23], Waardenburg syndrome [25], tuberous sclerosis complex [26]. However, there is a possibility that traditional methods for detecting CNVs cannot detect the low mosaic levels, so few pathogenic cases of low mosaic levels have been reported. Therefore, with LP GS, the study of pathogenicity of very low-level mosaicism is an interesting topic, which beyond the scope of this study.

In summary, the accuracy and the optimal sequencing depth of LP GS in the detection of mosaic aneuploidies and CNVs in prenatal diagnosis requires a comprehensive validation and evaluation. In this study, we first validated the accuracy of LP GS in the detection of mosaic aneuploidies and CNVs. Then we performed depth evaluation and found that the number of 30 M UAHRs (single-end 35 bp) was sufficient for the detection of mosaic aneuploidies and most mosaic CNVs with mosaic levels > 30%. This study could provide a reference for laboratories perform clinical LP GS in the detection of mosaic aneuploidies and CNVs.

### Electronic supplementary material

Below is the link to the electronic supplementary material.

Supplementary Material 1

Supplementary Material 2

Supplementary Material 3

## Data Availability

The data that support the findings of this study are not openly available due to reasons of sensitivity and are available from the corresponding author upon reasonable request.

## Abbreviations

LP GS Low: Pass genome sequencing
CNVs: Copy number variants
Mb: Megabases
UAHRs: Uniquely aligned high-quality reads
M: Million
FISH: Fluorescence in situ hybridization
QPCR: Quantitative fluorescent PCR
CMA: Chromosomal microarray analysis
DP: Number of UAHRs
DNBs: DNA NanoBalls
