# Supplementary material for: Accuracy and depth evaluation of clinical low pass genome sequencing in the detection of mosaic aneuploidies and CNVs
Source: BMC Med Genomics. 2023 Nov 17;16:294. doi: 10.1186/s12920-023-01703-8 (PMC10656965; doi:10.1186/s12920-023-01703-8)
Supplement: Supplementary file 1 — Supplementary Material 1 [file 12920_2023_1703_MOESM1_ESM.pdf]

Supplementary Methods

Comparative analysis for accuracy evaluation

To ensure that the accuracy evaluation was performed under a unified standard, the results at 35M uniquely aligned high-quality reads (UAHRs) were used as a standard for comparative analysis.

For simulated samples, the difference between the detected mosaic level and the theoretical mosaic level was calculated to evaluate the accuracy of LP GS in the detection of mosaic CNVs.

For virtual samples, the difference between the detected mosaic level and the mosaic level of Y chromosome was calculated to evaluate the accuracy of LP GS in the detection of mosaic aneuploidies and CNVs.

Calculation of mosaic levels for CNVs

For 3 down-sampling samples (35 M UAHRs) of each sample with CNVs at a certain theoretical mosaic level, true positives detected by LP GS were classified as mosaic CNVs with at least a 50% reciprocal overlap with the known positive region. If the number of true positives was 3, the median value of the 3 mosaic levels of true positives was taken as the detected mosaic level of the sample at 35 M UAHRs.

Calculation of mosaic levels for aneuploidies

For each down-sampling samples of each sample with aneuploidies at a certain theoretical mosaic level, first the largest overlap between the region detected by CNVseq and the known positive region was obtained, and $ratio_m$ of the largest overlap was used as a benchmark to select ratios ($ratio_i$) in the known positive region detected by CNVseq that met the following condition:

$$ratio_m - 0.1 \leq ratio_i \leq ratio_m + 0.1 \tag{1}$$

The average ratio (ave_{ratio}) was calculated with the following formula:

$$ave_{ratio} = \frac{\sum_1^n ratio_i * region_i}{\sum_1^n region_i} \tag{2}$$

Where n is the number of regions that meet the condition (1) and $region_i$ is the length of the region corresponding to $ratio_i$ that meets the condition (1).

The mosaic levels of aneuploidies were then estimated by the average ratio (ave_{ratio}). The median value of the 3 mosaic levels of 3 down-sampling samples (35 M UAHRs) was taken as the detected mosaic level of the sample at 35 M UAHRs.

Calculation of mosaic levels for Y chromosome

For each down-sampling samples of each sample at a certain theoretical mosaic level, first the largest overlap between the region detected by CNVseq and the Y chromosome was obtained, and $ratio_{m_y}$ of the largest overlap was used as a benchmark to select ratios ($ratio_j$) in the Y chromosome detected by CNVseq that met the following condition:

$$ratio_{m_y} - 0.05 \le ratio_j \le ratio_{m_y} + 0.05 \quad (3)$$

The average ratio (ave_{ratio_y}) was calculated with the following formula:

$$ave_{ratio_y} = \frac{\sum_1^{n_y} ratio_j * region_j}{\sum_1^{n_y} region_j} \quad (4)$$

Where n_y is the number of regions that meet the condition (3) and $region_j$ is the length of the region corresponding to $ratio_j$ that meets the condition (3).

The mosaic levels of the Y chromosome were then estimated by the average ratio (ave_{ratioy}). The median value of the 3 mosaic levels of 3 down-sampling samples (35 M UAHRs) was taken as the detected mosaic level of the sample at 35 M UAHRs.

Sensitivity statistics for depth evaluation

To evaluate the influence of sequencing depth on LP GS in the detection of mosaic aneuploidies and CNVs, UAHRs were used to generate downsampling samples for 6 mosaic aneuploidies and 15 mosaic CNVs in virtual samples. The results of LP GS in downsampling samples at a certain theoretical mosaic level were compared with those of LP GS using 35 M UAHRs at the same theoretical mosaic level.

For each down-sampling sample at a certain theoretical mosaic level, true positives detected by a certain number of UAHR were classified as mosaic CNVs that met the

following conditions: 1) mosaic CNVs detected after down-sampling with at least a 50% reciprocal overlap with known positive regions and confirmed by visualization of the copy ratio using an in-house script; 2) the difference between the mosaic levels estimated after down-sampling and the mosaic levels (or the mosaic levels of Y chromosome if CNVseq failed to detect the mosaic levels using 35 UAHRs) estimated using 35 M UAHRs was ≤ 3%.

For each down-sampling sample at a certain theoretical mosaic level, true positives detected by a certain number of UAHR were classified as mosaic aneuploidies that met the following condition: the difference between the mosaic levels estimated after down-sampling and the mosaic levels estimated using 35 M UAHRs was ≤ 3%.

With a certain UAHR, the detection sensitivity for each mosaic level interval ($Sensitivity_{mosaic\ level\ interval}$) was calculated by the following formula:

$$Sensitivity_{mosaic\ level\ interval} = \frac{N_{true\ positives}}{N_{CNVs}*12} \quad (5)$$

N_{CNVs} is the number of mosaic aneuploidies and CNVs with mosaic levels in a mosaic interval. $N_{true\ positives}$ is the number of true positives with mosaic levels in the mosaic level interval detected by a certain number of UAHR.

With a certain UAHR, the total detection sensitivity for all mosaic level intervals ($Sensitivity_{total}$) was calculated by the following formula:

$$Sensitivity_{total} = \frac{N_{total\ true\ positives}}{N_{total}*12} \quad (6)$$

N_{total} is the number of all mosaic aneuploidies and CNVs. Where $N_{total\ true\ positives}$ is the number of all true positives detected by a certain number of UAHR.
