# Supplementary material for: Accuracy and depth evaluation of clinical low pass genome sequencing in the detection of mosaic aneuploidies and CNVs
Source: BMC Med Genomics. 2023 Nov 17;16:294. doi: 10.1186/s12920-023-01703-8 (PMC10656965; doi:10.1186/s12920-023-01703-8)
Supplement: Supplementary file 3 — Supplementary Material 3 [file 12920_2023_1703_MOESM3_ESM.docx]

**Supplementary figure S1**


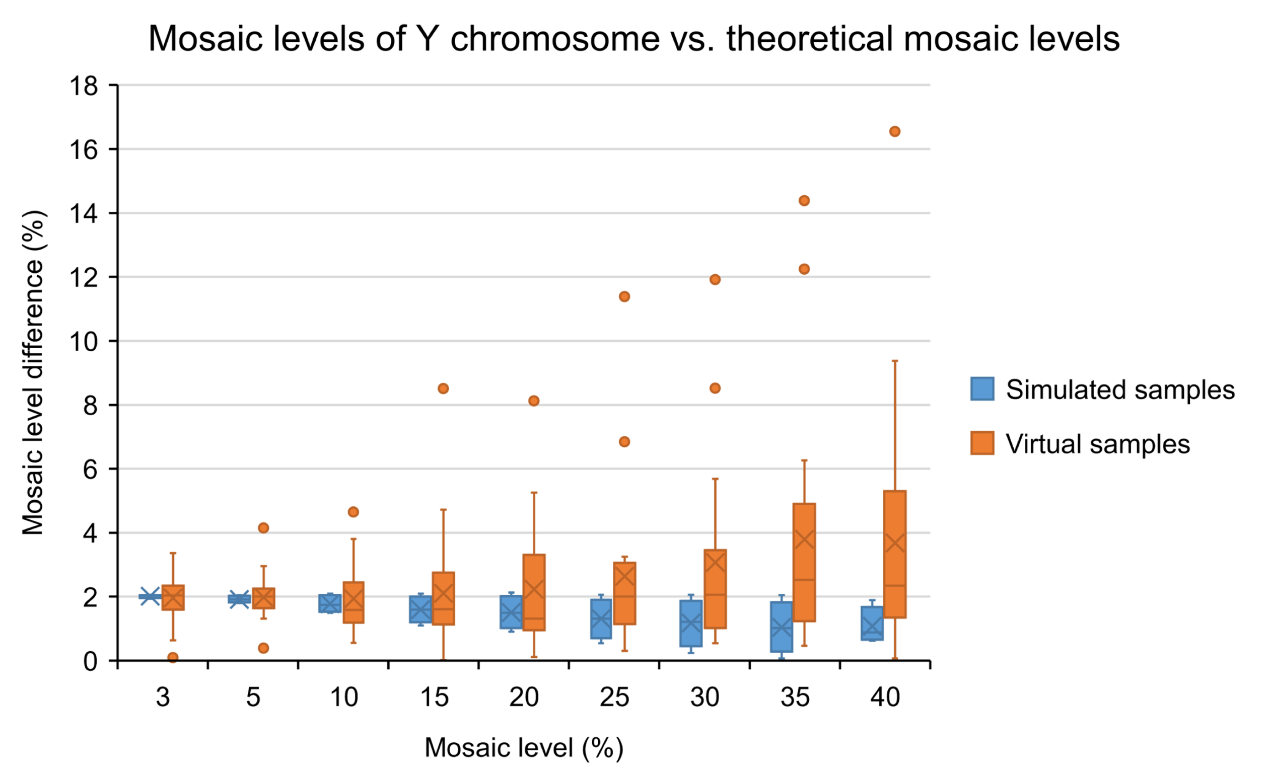


Supplementary figure S1. Comparative analysis of the mosaic levels of Y chromosome and the theoretical mosaic levels in simulated samples and virtual samples. We compared the differences between the mosaic levels of Y chromosome and the theoretical mosaic levels in simulated samples and virtual samples, and found that the differences between the mosaic levels of Y chromosome and the theoretical mosaic levels fluctuated greatly in virtual samples (Figure S1).
